# Risk stratification of LA‐NPC during chemoradiotherapy based on clinical classification and TVRR

**DOI:** 10.1002/cam4.7029

**Published:** 2024-02-23

**Authors:** Qianlong Tang, Chaorong Mei, Bei Huang, Rui Huang, Le Kang, Ailin Chen, Na lei, Pengcheng Deng, Shouyan Ying, Peng Zhang, Yuan Qin

**Affiliations:** ^1^ Department of Oncology Sichuan Mianyang 404 Hospital, First People's Hospital of Mianyang Mianyang China; ^2^ Hospital of Chengdu Office of People's Government of Tibetan Autonomous Region (Hospital.C.T.) Chengdu China; ^3^ Department of Oncology Third People's Hospital of Mianyang Mianyang China; ^4^ Department of Radiation Oncology, Sichuan Cancer Hospital and Institute, Sichuan Cancer Center, School of Medicine University of Electronic Science and Technology of China, Radiation Oncology Key Laboratory of Sichuan Province Chengdu China; ^5^ Department of Hematology and Oncology Anyue County People's Hospital Ziyang China; ^6^ West China Tianfu Hospital ,Sichuan University Chengdu China; ^7^ Department of Oncology Chengdu Qingbaijiang District People's Hospital Chengdu China; ^8^ Department of Oncology Yunnan Cancer Hospital Kunming China

**Keywords:** clinical typing, nasopharyngeal carcinoma, prognosis, risk stratification, tumor volume reduction rate

## Abstract

**Purpose:**

To investigate the correlation between tumor volume reduction rate (TVRR) and prognosis in patients with diverse clinical types of nasopharyngeal carcinoma (NPC) undergoing chemoradiotherapy, thereby aptly categorizing risks and directing the personalized treatment of NPC.

**Materials and Methods:**

A total of 605 NPC patients with varying clinical types were enrolled in this study and subsequently segregated into six subgroups based on their clinical types and TVRR. To accentuate the efficacy of grouping, Groups 1–6 underwent clustered analysis of hazard atio (HR) values pertaining to progression‐free survival (PFS), forming three risk clusters denoted as low, intermediate, and high. The log‐rank test was employed to discern differences, and R 4.1.1 was utilized for cluster analysis.

**Results:**

According to survival rates, we classified the first (G2 and G4), second (G1 and G6), and third (G3 and G5) risk clusters as low‐, intermediate‐, and high‐risk, respectively. When comparing risk stratification with the 8th edition of the TNM staging system, our classification exhibited superior predictive prognostic performance. Subgroup analysis of treatments for each risk cluster revealed that the PFS in the neoadjuvant chemotherapy (NACT) + concurrent chemoradiotherapy (CCRT) group surpassed that of the CCRT group significantly (*p* < 0.05).

**Conclusion:**

The reliance on clinical types and TVRR facilitates risk stratification of NPC during chemoradiotherapy, providing a foundation for physicians to tailor therapeutic strategies. Moreover, the risk cluster delineated for NPC patients during the mid‐term of chemoradiotherapy stands as an independent prognostic factor for progression‐free survival (PFS), overall survival (OS), distantmetastasis‐free survival (DMFS), and local recurrence‐free (LRRFS) posttreatment. Additionally, individuals in the high‐risk cluster are recommended to undergo adjuvant chemotherapy after CCRT.

## Graphical abstract

This work investigated the correlation between tumor volume reduction rate (TVRR) and prognostic value in 605 cases with different clinical stages of locally advanced nasopharyngeal carcinoma during chemoradiotherapy. Our results indicated that clinical types and TVRR can carry out risk stratification for nasopharyngeal carcinoma (NPC) during chemoradiotherapy, enabling clinicians to assess NPC patients with poor therapeutic effect and high risk of failure after treatment, and providing a basis for adjusting therapeutic regimen. In addition, NPC patients in the high‐risk cluster are allowed to take adjuvant chemotherapy after concurrent chemoradiotherapy.

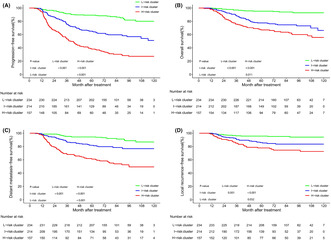

## 1 INTRODUCTION

Nasopharyngeal carcinoma (NPC) represents a malignancy arising from the epithelium of the nasopharyngeal mucosa, with its incidence intricately linked to genetic susceptibility, environmental factors, and Epstein–Barr virus (EBV) infection. According to the global cancer statistics of 2020, approximately 133,354 newly diagnosed with NPC and 80,008 NPC‐related fatalities worldwide.^1^ Owing to its distinctive biological attributes, insidious onset, and pronounced invasiveness in local domains, a majority of patients are diagnosed with locally advanced nasopharyngeal carcinoma (LA‐NPC) during their first hospital visit. Despite adherence to standard treatment protocols, the risk of local recurrence and distant metastasis persists, with the latter manifesting as the predominant concern.^2^, ^3^, ^4^ Currently, the prognosis evaluation of NPC primarily relies on the American Joint Committee on Cancer (AJCC)/International Union for Cancer Control (UICC) Lymph Node Metastasis (TNM) staging system.^5^ Nevertheless, individuals with NPC sharing identical TNM stages and treatment regimens still exhibit markedly disparate survival outcomes.^6^ To promptly identify patients facing inferior treatment prognoses, risk assessments must be carried out during treatment, facilitating the expeditious modification of treatment decisions for those with high‐risk LA‐NPC.

According to the tumor's biological behavior of NPC, LA‐NPC can be delineated into three distinct clinical types,^7^ accentuating the inherent progression characteristics of nasopharyngeal tumors. First, denominated the ascending type, predominantly manifests as a locally advanced lesion (T3‐4), concomitant with the presence of cervical lymph nodes in the initial stages (N0‐1), predisposing it to local recurrence. Second, termed the descending type, primarily displays cervical lymph node lesions (N2‐3) and early‐phase lesions (T1‐2), rendering it susceptible to distant metastasis. Third, known as the mixed type, represents advanced local disease (T3‐4) coupled with advanced cervical lymph node lesions (N2‐3), constituting the highest likelihood of both local recurrence and distant metastasis. Research has confirmed significant differences in the prognosis among the various clinical types of NPC,^8^, ^9^ indicating that distinct clinical types may furnish supplementary biological insights to the TNM staging system, thereby aiding in prognostic predictions for LA‐NPC. Concurrent chemoradiotherapy (CCRT) with or without neoadjuvant chemotherapy (NACT) stands as the primary therapeutic modality for LA‐NPC. Nevertheless, even with the administration of NACT plus CCRT, approximately 20% of LA‐NPC patients encounter treatment failure.^10^, ^11^ In pursuit of heightened survival rates, investigators have delved into the relationship between tumor chemoradiation sensitivity and prognosis. Notably, it has been documented that the tumor volume reduction rate (TVRR) serves as an autonomous prognostic factor in radical radiotherapy for NPC.^12^, ^13^

Based on the aforementioned foundations, both the clinical classifications of NPC and the TVRR emerge as pivotal prognostic indicators, holding the potential to furnish robust supplementary insights for the TNM staging system. In this context, these two factors to execute risk stratification during patient treatment could potentially enhance the therapeutic outcomes for NPC. Consequently, we undertook a study encompassing NPC patients treated at the Sichuan Cancer Hospital, aiming to ascertain the collective prognostic value of diverse clinical types of NPC and the tumor regression rate during the mid‐term of chemoradiotherapy. Our objective was to promptly identify individuals with NPC exhibiting poor treatment responses and a heightened risk of posttreatment failure. This approach aims to guide the personalized treatment of patients grappling with LA‐NPC in a more efficacious manner.

## 2 MATERIALS AND METHODS

### 2.1 Research subjects

Patients with pathologically confirmed NPC and devoid of distant metastases at the Sichuan Cancer Hospital between January 2010 and December 2016 were enrolled. This study was approved by the Ethics Committee of Medical Research and New Medical Technology of Sichuan Cancer Hospital (SCCHEC‐02‐2022‐160). Inclusion criteria comprised: (1) patients diagnosed with stages III‐IVa disease according to the eighth edition of the American Cancer Society. (2) Individuals undergoing radical chemoradiotherapy, along with concurrent or adjuvant chemotherapy in our hospital. (3) Patients subjected to enhanced CT (computerized tomography) and MRI (magnetic resonance imaging) of the nasopharynx and neck both before treatment and finished the radiotherapy for 20 fractures. (4) Those receiving no anti‐tumor therapy during their initial visit to our hospital. (5) Individuals devoid of additional malignant tumors or severe cardiac, pulmonary, hepatic, and renal afflictions. Exclusion criteria incorporated: (1) Patients presenting with distant metastases at the first visit. (2) Individuals afflicted with concurrent malignant tumors or profound organic lesions. (3) Patients lacking enhanced CT or MRI images of the nasopharynx and neck, either pre‐treatment or throughout the therapeutic course.

### 2.2 Therapeutic modalities

In this study, the therapeutic protocol for LA‐NPC was concurrent radiochemotherapy CCRT ± NACT or CCRT ± adjuvant chemotherapy (ACT). The two‐drug regimen, TP regimen based on platinum, was employed for both NACT and ACT. The ‘T’ designates paclitaxel (135 mg/m^2^, d1)/docetaxel (75 mg/m^2^, d1), and ‘P’ signifies cisplatin (25 mg/m^2^, d1‐3)/Nedaplatin (80 mg/m^2^, d1). Administration occurred once every 3 weeks for a total of 2–3 courses. The concurrent chemotherapy regimen was comprised a platinum‐based single/dual‐drug regimen, specifically TP or P regimen, with one course applied every 3 weeks for a total of 2 courses. In our study, if patients with severe myelosuppression, the dose of the original treatment regimen was adjusted by 80%, but the treatment regimen was not adjusted. The radiotherapy target area adhered to the guidelines outlined in the No. 50 and No. 62 reports of the International Commission on Radiological Units and Measurements (ICRU).^14^ The radiotherapy dose and the threshold dose for organs at risk, including the spinal cord, brainstem, optic nerve/chiasm, and temporal lobe, adhered to the relevant regulations of the United States Radiotherapy Cooperative Group (RTOG‐0615, RTOG‐0225). For conventional fractionation, the dose was maintained at 2.10–2.25G y/f, with the accumulated dose of pv‐GTV was about 66–76G y. The total prescribed doses for pv‐GTVln, pv‐CTV1 (low‐risk region), and pv‐CTV2 (high‐risk region) were 66–70G y, 60–66G y, and 54–60G y, respectively, administered once daily, five times a week.

### 2.3 Clinical stage and types

Referring to the 8th edition of the UICC/AJCC staging system, we re‐staged the enrolled patients based on the completed systemic examination data conducted before treatment.^15^ Predicated on the tumor's biological behavior, LA‐NPC was divided into three distinct clinical types: ascending type (type A), descending type (type D), and mixed type (type AD). The ascending type corresponded to type A NPC (T3‐4N0‐1M0); the descending type corresponded to type D NPC (T1‐2N2‐3M0); and the mixed type corresponded to type AD NPC (T3‐4N2‐3M0).

### 2.4 Extraction of tumor volume and calculation of TVRR

MRI and CT examinations were conducted twice for all patients, initially before any treatment and subsequently after the completion of 20 fractions radiation therapy. The image fusion functionality of the MIM(Medical Image Merge) software system facilitated the registration of the obtained MRI and CT images. The gross tumor volume (GTV) comprised the gross tumor volume of nasopharyngeal carcinoma (GTVnx) and the gross tumor volume of the lymph node (GTVln). The delineation of GTV primarily relied on the visualization provided by the fusion images. In instances of disagreement regarding the delineation of GTV, a physician specializing in head and neck radiotherapy and another radiologist specializing in head and neck imaging collaboratively determined the tumor boundary. Volume parameters of the selected delineation were automatically calculated and extracted through the MIM system's target area delineation workstation. The total volumes before treatment and during chemoradiotherapy were designated as PGTV (cm^3^) and MGTV (cm^3^), respectively. The TVRR was calculated as follows: TVRR = (PGTV−MGTV)/PGTV × 100%. Furthermore, we defined PFS as the endpoint using the ROC curve and the Youden index. Subsequently, the optimal cut‐off for TVRR was determined to be 50%.

### 2.5 Definition of subgroups by clinical types and TVRR

**FIGURE 1:**
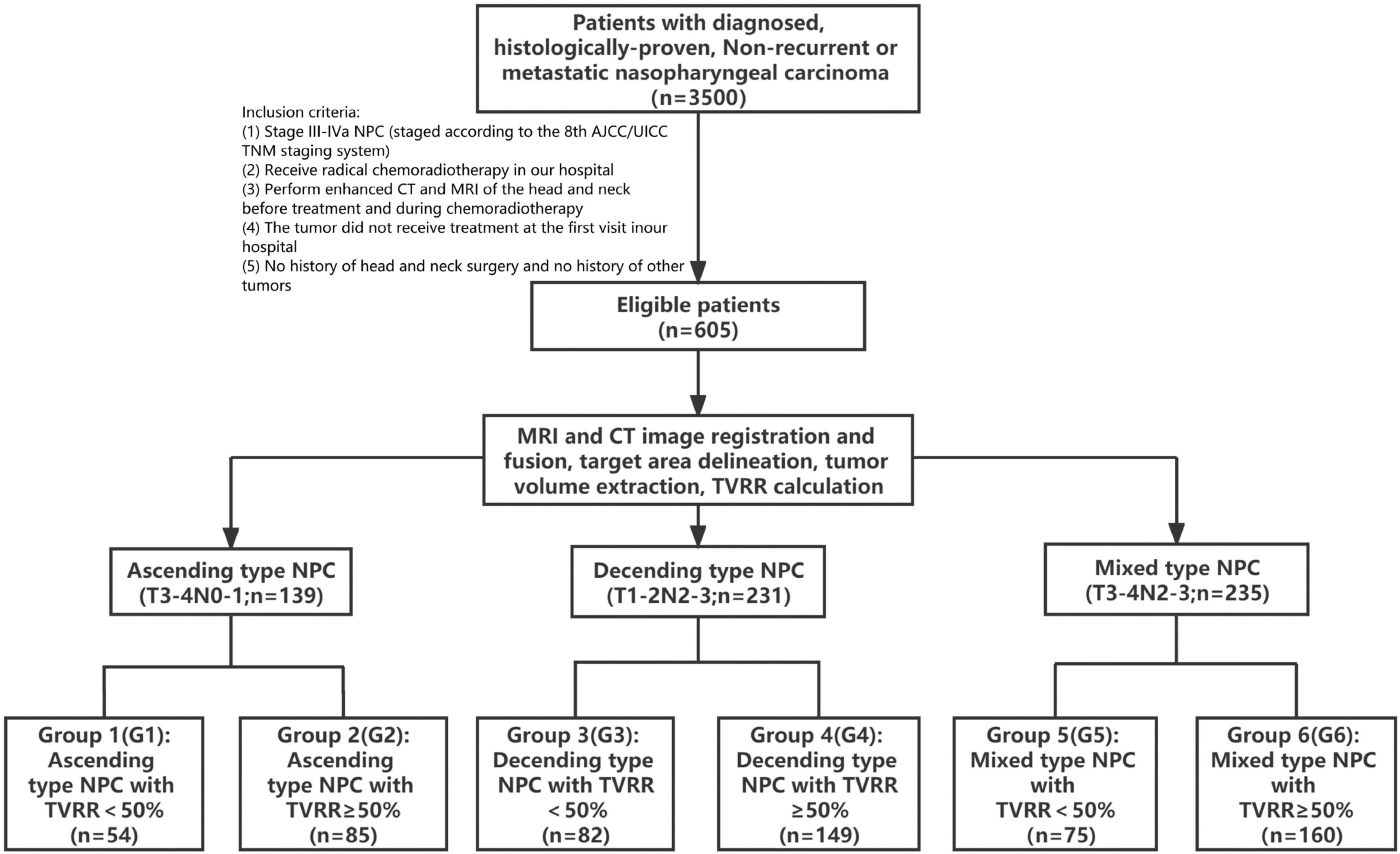
Flow chart. NPC; nasopharyngeal carcinoma.

All enrolled NPC patients were classified into 6 groups according to different clinical types (type A, D, and AD) and the level of TVRR: Group 1 (G1) denoted type A with TVRR <50%; Group 2 (G2) denoted type A with TVRR ≥50%; Group 3 (G3) denoted type D with TVRR <50%; Group 4 (G4) denoted type D with TVRR ≥50%; Group 5 (G5) denoted AD type with TVRR <50%; and Group 6 (G6) denoted AD type with TVRR ≥50%. The delineation of this process is depicted in Figure 1.

### 2.6 Follow‐up and clinical endpoint

All patients underwent regular follow‐ups after the completion of their respective treatments. The follow‐up schedule comprised assessments every 3 months during the initial 2 years following the conclusion of treatment, every 6 months for the subsequent 2–5 years, and subsequently, annual evaluations beyond the 5‐year mark until the occurrence of death. The follow‐up assessments encompassed interrogations, physical examinations, blood routine analyses, blood biochemistry assessments, thyroid function, tumor markers, nasopharyngeal endoscopy, head and neck MRI, chest CT, and abdominal ultrasound examinations. The primary endpoint of this study was PFS, and the secondary endpoints were OS, DMF, and LRRFS.

### 2.7 Statistical analysis

The statistical analysis was executed using SPSS 26.0 and R 4.1.1. All statistical tests were two‐tailed, and significance was set at *p* < 0.05. Survival outcomes were calculated utilizing the Kaplan–Meier method, and the Log‐rank method was employed to compare survival results. Risk stratification was implemented through K‐means clustering, based on the relative between‐group HR values for PFS. Cox proportional hazard models were utilized for univariate and multivariate analyses, scrutinizing the defined risk clusters and exploring the correlation between clinical features and survival rate. The consistency index (C‐index) and the area under the ROC curve (AUC value) were utilized to evaluate the predictive efficacy of both the TNM staging system and the risk stratification.

## 3 RESULTS

### 3.1 General information

A total of 605 NPC patients in stages III‐IVa were enrolled in this study, with a median follow‐up duration of 70.7 months (4.8–120 months). Detailed clinical and treatment characteristics for the three clinical types of NPC are provided in Table 1.

**TABLE 1:** Clinical characteristics of patients in three clinical types of NPC.

Characteristic	Type A total data *n* = 139	Type D total data *n* = 231	Type AD total data *n* = 235	χ^2^	*p* value^a^
Sex				1.159	0.560
Male	97 (69.8%)	162 (70.1%)	174 (74.0%)		
Female	42 (30.2%)	69 (29.9%)	61 (26.0%)		
Age (years)				1.932	0.381
<50	72 (51.8%)	136 (58.8%)	136 (57.8%)		
≥50	67 (48.2%)	95 (41.2%)	99 (42.2%)		
Overall stage (^b^ 8th edition)				84.17	<0.001
III	53 (38.1%)	164 (70.9%)	71 (30.2%)		
IVa	86 (61.9%)	67 (29.1%)	164 (69.8%)		
Smoking				0.914	0.633
Yes	59 (42.4%)	88 (38.0%)	98 (42.8%)		
No	80 (57.6%)	143 (61.9%)	137 (58.2%)		
Alcoholism				0.812	0.666
Yes	35 (25.2%)	49 (21.2%)	55 (23.4%)		
No	104 (74.8%)	182 (78.8%)	180 (76.6%)		
Family of cancer history				1.238	0.538
Yes	15 (10.8%)	23 (9.9%)	18 (7.7%)		
No	124 (89.2%)	208 (90.1%)	217 (92.3%)		
T staging (8th edition)				606.4	<0.001
T1	0 (0.0%)	29 (12.5%)	0 (0.0%)		
T2	0 (0.0%)	202 (87.5%)	0 (0.0%)		
T3	53 (38.1%)	0 (0.0%)	101 (42.9%)		
T4	86 (61.9%)	0 (0.0%)	134 (57.1%)		
N staging (8th edition)				606.6	<0.001
N0	16 (11.5%)	0 (0.0%)	0 (0.0%)		
N1	123 (88.5%)	0 (0.0%)	0 (0.0%)		
N2	0 (0.0%)	162 (70.1%)	175 (74.4%)		
N3	0 (0.0%)	69 (29.9%)	60 (25.6%)		
TVRR				1.454	0.483
<50%	46 (33.1%)	69 (29.8%)	64 (27.2%)		
≥50%	93 (66.9%)	162 (70.2%)	171 (72.8%)		
HGB level, g/L				2.502	0.286
<120	17 (12.2%)	17 (7.3%)	21 (8.9%)		
≥120	122 (87.8%)	214 (92.7%)	214 (91.1%)		
CRP level, g/mL				2.161	0.339
<8	127 (91.4%)	211 (91.3%)	206 (87.6%)		
≥8	12 (8.6%)	20 (8.7%)	29 (12.4%)		
ALT, U/L				0.728	0.694
<24	73 (52.5%)	111 (48.1%)	115 (48.9%)		
≥24	66 (47.5%)	120 (51.9%)	120 (50.1%)		
AST, U/L				0.295	0.862
<23	67 (48.2%)	105 (45.4%)	111 (47.2%)		
≥23	72 (51.8%)	126 (54.6%)	124 (52.8%)		
ALB, g/L				7.382	0.025
<40	18 (12.9%)	17 (7.3%)	36 (15.4%)		
≥40	121 (87.1%)	214 (92.7%)	199 (84.6%)		
LDH level, U/L				1.792	0.408
<180	89 (64.0%)	132 (57.1%)	143 (60.8%)		
≥180	50 (36.0%)	99 (42.9%)	92 (39.2%)		
NLR				4.613	0.099
<2.26	58 (41.7%)	111 (48.1%)	90 (38.2%)		
≥2.26	81 (58.3%)	120 (51.9%)	145 (61.8%)		
PLR				0.284	0.867
<88	30 (21.6%)	49 (21.2%)	46 (19.5%)		
≥88	109 (78.4%)	182 (78.8%)	189 (80.5%)		
PNI				0.319	0.825
<52	75 (54.0%)	122 (58.8%)	120 (51.0%)		
≥52	64 (46.0%)	109 (41.2%)	115 (49.0%)		
Treatment modality				6.726	0.151
NACT plus CCRT	73 (52.5%)	135 (58.4%)	110 (46.8%)		
CCRT alone	47 (33.8%)	67 (29.1%)	92 (39.1%)		
CCRT plus ACT	19 (13.7%)	29 (12.5%)	33 (14.1%)		

*Note*: Ascending type NPC: NPC patients with stage T3–4N0–1; Descending type NPC: NPC patients with stage T1–2N2–3; Mixed type NPC: NPC patients with stage T3–4N2–3; Statistical comparisons between three types NPC were computed using the Chi‐squared test.

Abbreviations: ACT, adjuvant chemotherapy; ALB, albumin; CCRT, concurrent chemoradiotherapy; CRP, C‐reactive protein; HGB, hemoglobin; LDH, lactate dehydrogenase; NACT, neoadjuvant chemotherapy; NLR, neutrophil‐lymphocyte ratio; PLR, platelet‐lymphocyte ratio; PNI, prognostic nutritional index; TVRR, tumor volume reduction rate during chemoradiotherapy.

^a^ Clinicopathology and treatment characteristics were compared using the χ^2^ test, or Fisher's exact test, *p*<0.05 indicates a significant difference.

^b^ According to the 8th edition of the AJCC/UICC staging system.

### 3.2 Survival analysis of G1–6 subgroups

The 5‐year PFS, OS, DMFS, and LRRFS values for the G1–6 subgroups are presented in Table S1. While not all subgroup survival curves exhibited significant separation (S2), G3 and G5 displayed the lowest survival rates, G2 and G4 showed the highest survival rates, and G1 and G6 demonstrated superior survival rates compared to G3 and G5.

### 3.3 Construct risk stratification to predict prognosis

**FIGURE 2:**
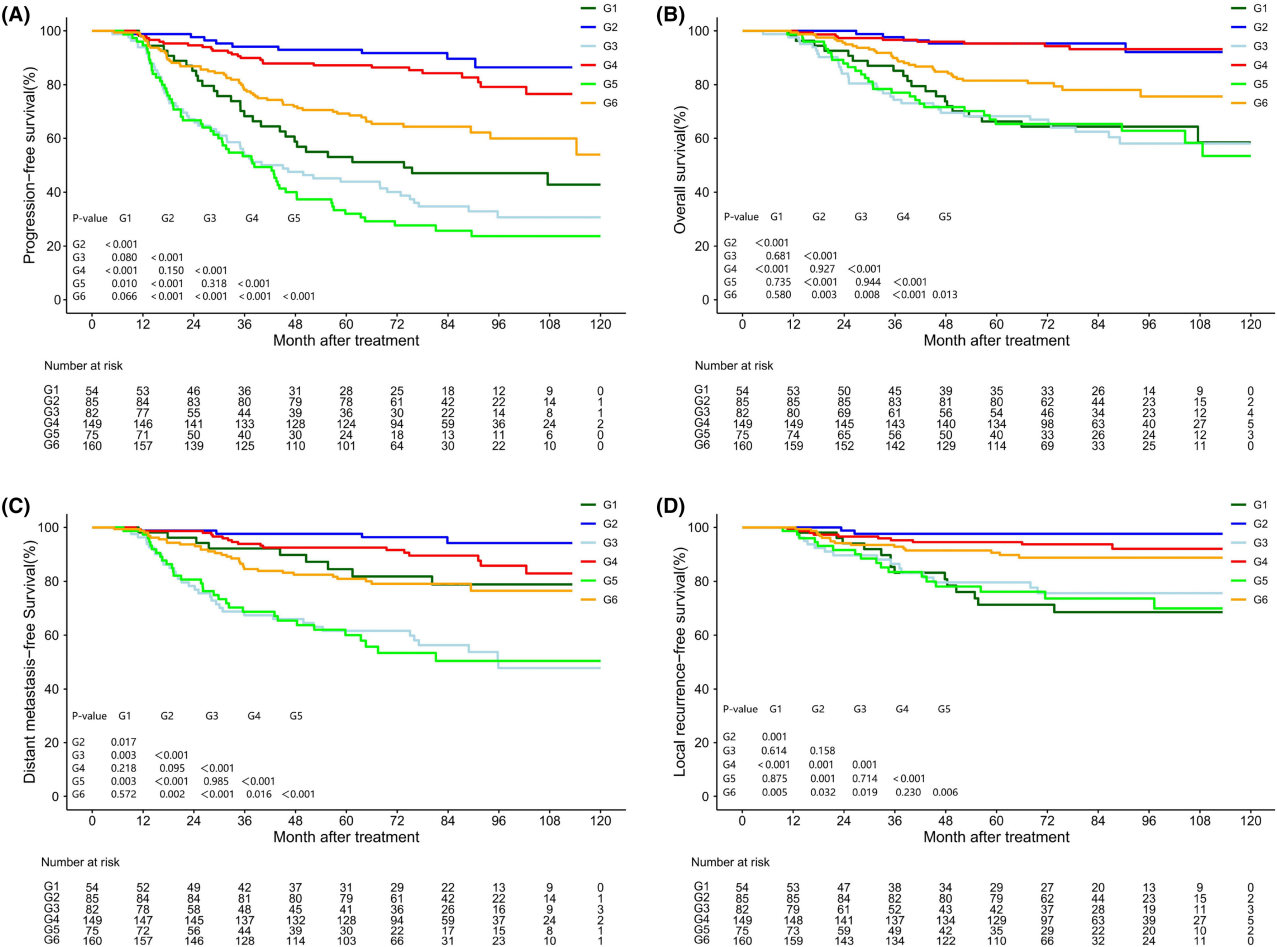
(A) Kaplan–Meier plots of survival outcomes for locally advanced nasopharyngeal carcinoma patients in six subgroups stratified by locoregional extension patterns combined with tumor volume reduction rate. The results are PFS (A), OS (B), DMFS (C), and LRRFS (D).

**FIGURE 3:**
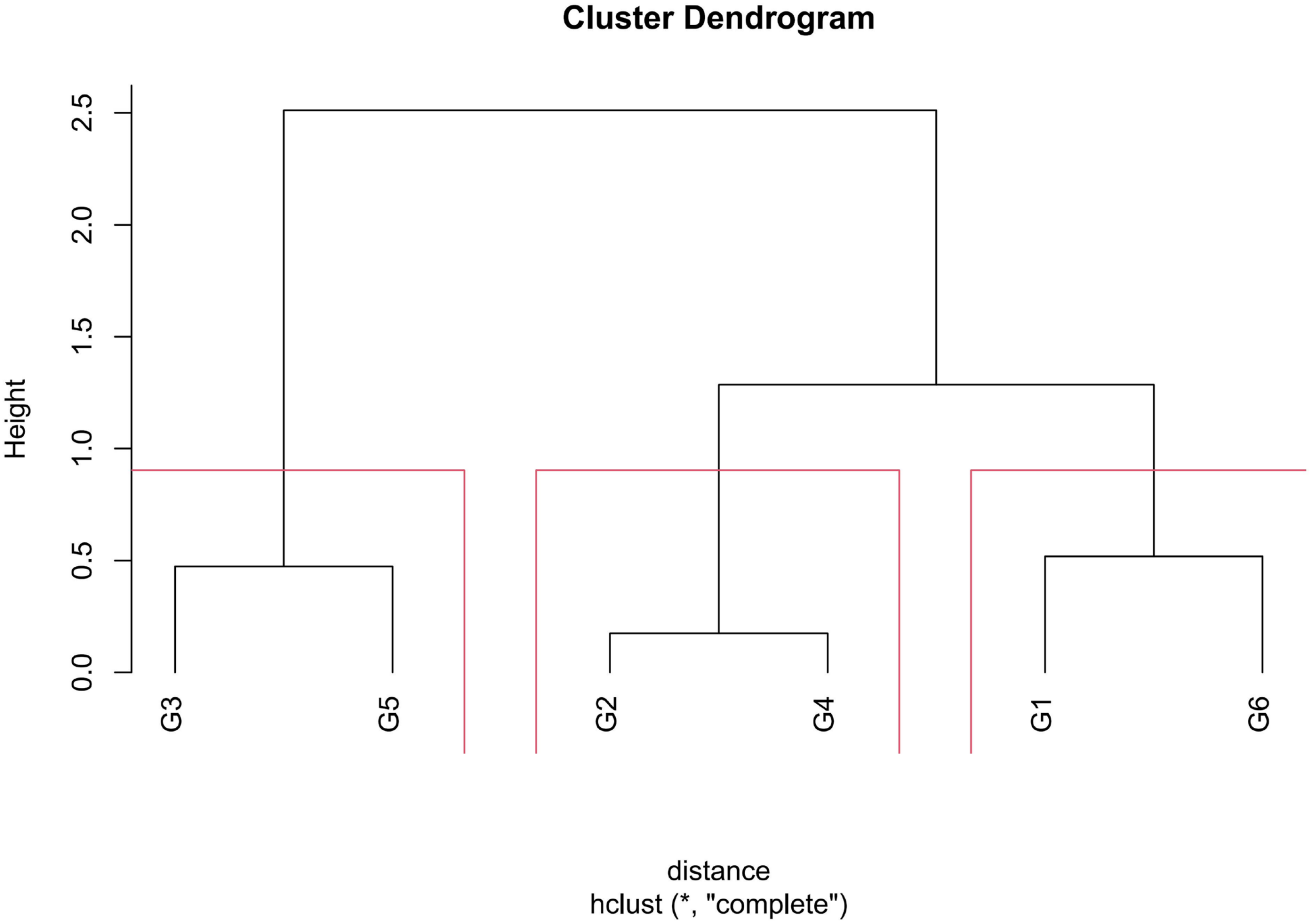
Results of K‐means cluster analysis for subgroups G1–6. G1–G6 were clustered into three risk clusters using a supervised clustering approach. G2 and G4 were assigned to risk cluster 1 (low‐risk cluster); G1 and G6 were assigned to risk cluster 2 (intermediate‐risk cluster); G3 and G5 were assigned to risk cluster 3 (high‐risk cluster).

**FIGURE 4:**
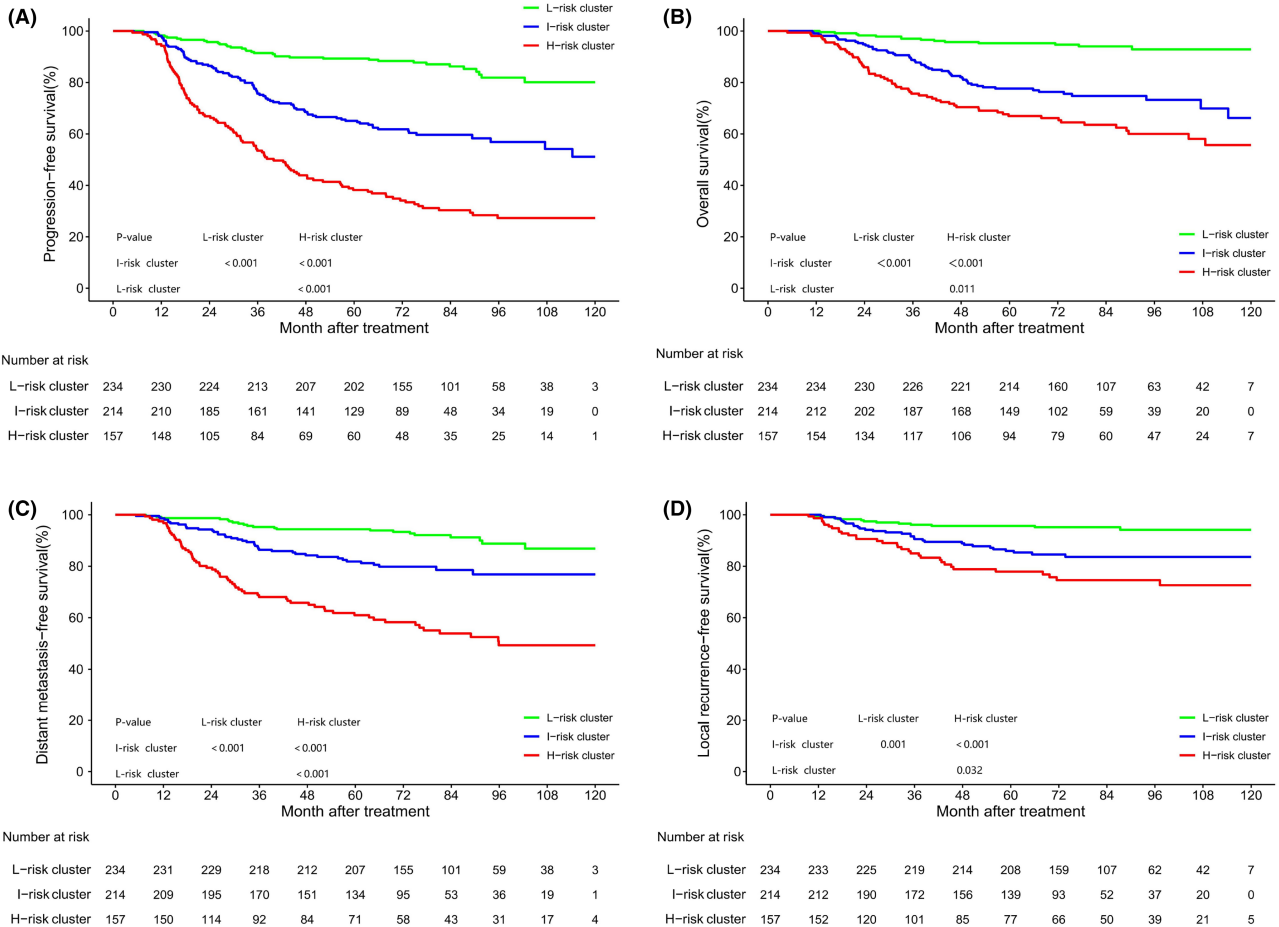
Kaplan–Meier plots of survival outcomes for patients with locally advanced nasopharyngeal carcinoma in three distinct risk clusters. Results are shown for PFS (A), OS (B), DMFS (C), and LRRFS (D).

As displayed in Figure 2, there were overlaps among the survival curves for G1–G6 prompting a cluster analysis based on the relative HR for PFS. This analysis identified three distinct risk clusters (Figure 3) categorized as low‐, intermediate‐, and high‐risk based on the survival rates of PFS, OS, DMFS, and LRRFS. The survival curves for the three risk clusters are illustrated in Figure 4, with incident numbers provided in Table S2. The results of COX multivariate analysis indicated that the risk cluster was an independent prognostic factor for NPC (Figure S1).

### 3.4 Risk stratification system compared with TNM staging system

**FIGURE 5:**
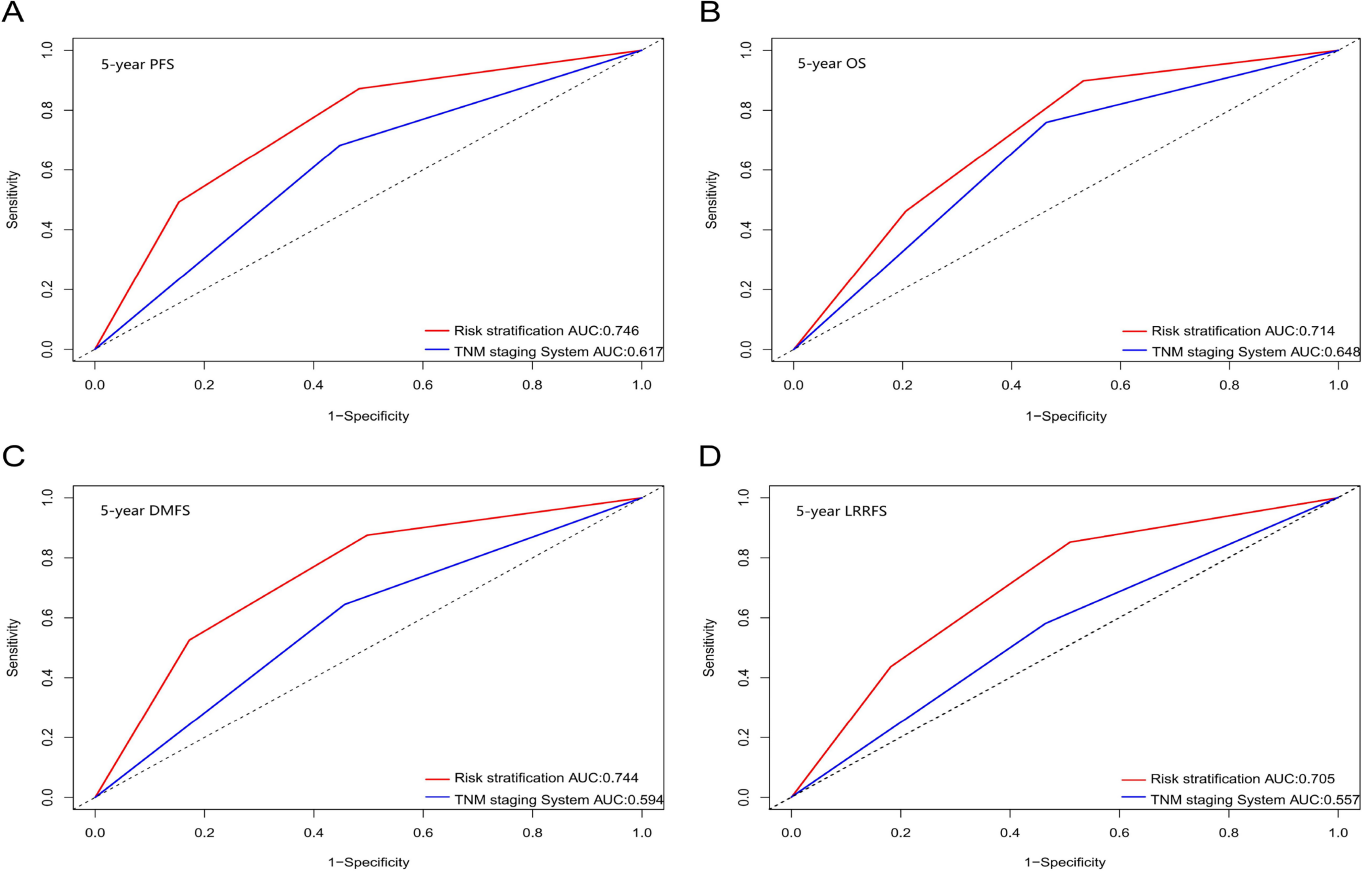
The comparison of prognostic performance between risk stratification system and TNM staging system. Results are shown for 5‐year PFS (A), 5‐year OS (B), 5‐year DMFS (C), and 5‐year LRRFS (D).

Evaluation of the prognostic performance of the risk cluster and the TNM staging system, based on consistency C‐index scores and the area under the curve (ROC) AUC values, revealed that the C‐index of the risk cluster surpassed that of the TNM staging system (Table S4). Additionally, the AUC value for the risk cluster exceeded that of the TNM staging system (Figure 5), indicating superior prognostic prediction performance.

### 3.5 Individualized treatment strategies based on risk stratification

**FIGURE 6:**
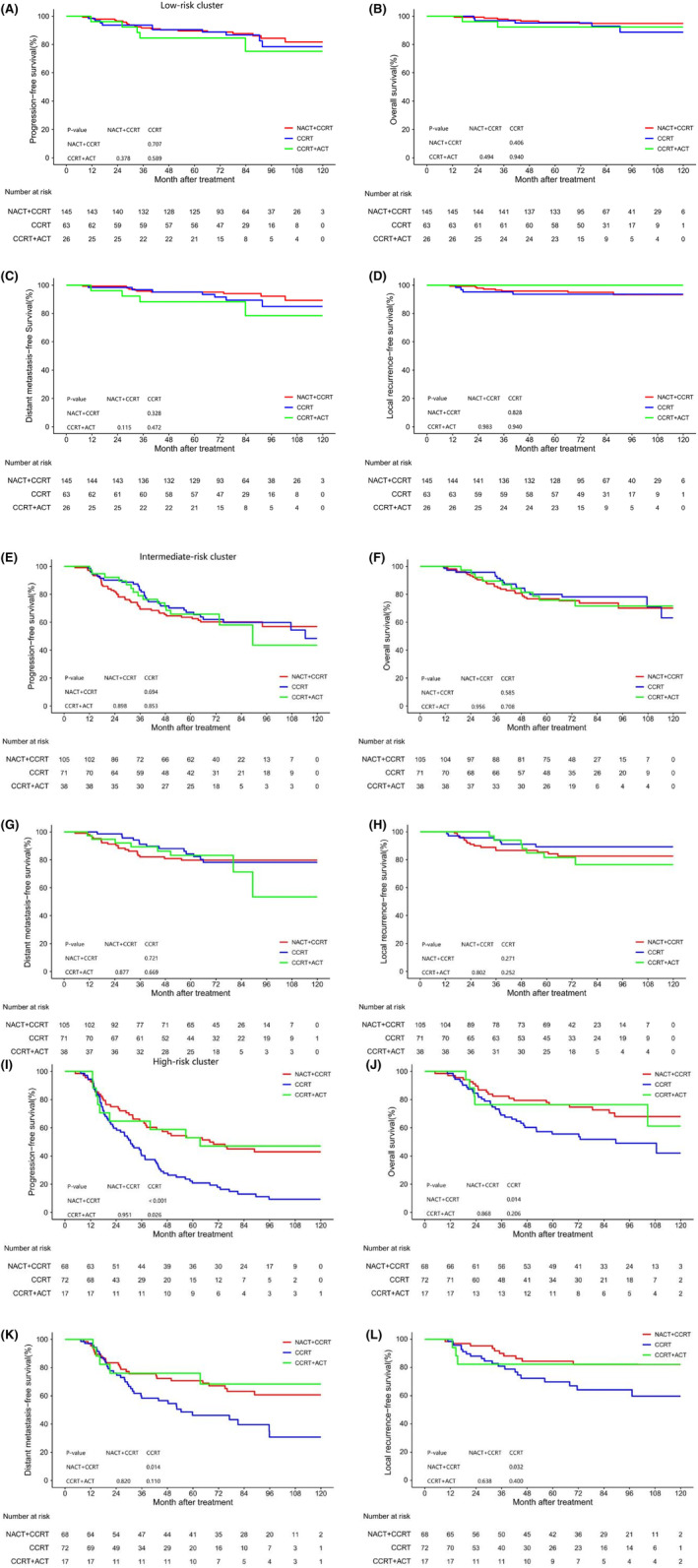
Survival analysis of subgroups with different treatments. Results are shown for low‐risk cluster (A‐D), intermediate‐risk cluster (E‐H), and high‐risk cluster (I‐L).

By comparing the efficacy of three treatment options (NACT+CCRT, CCRT, CCRT+ACT), optimal therapeutic regimens for each risk cluster were discerned (Figure 6). In the low‐ and intermediate‐risk clusters, survival curves for the three treatment methods showed similar effects without statistically significant differences (*p* > 0.05). However, within the high‐risk cluster, NACT+CCRT and CCRT+ACT demonstrated superiority over CCRT, with statistically significant differences (*p* < 0.05). Notably, NACT+CCRT and CCRT+ACT exhibited comparable efficacy. Multivariate analysis within the high‐risk cluster underscored the therapeutic regimen as an independent prognostic factor for PFS, OS, DMFS, and LRRFS (Figure S2).

## 4 DISCUSSION

This study enrolled a total of 605 LA‐NPC patients who underwent radical chemoradiotherapy at Sichuan Cancer Hospital. According to the clinical types and mid‐term TVRR of chemoradiotherapy, the included patients were divided into six groups, namely G1‐6. Subsequent survival analyses were executed on these cohorts. The obtained HR values for PFS were analyzed by K‐means clustering, resulting in the identification of low‐, intermediate‐, and high‐risk clusters. Notably, we observed that the survival curves between risk clusters presented a good degree of discrimination, and the survival curves of PFS, OS, DMFS, and LRRFS in G1–6 groups had more obvious separation and stronger discrimination performance. A comparative analysis with the TNM staging system revealed that the risk cluster displayed a notably superior predictive performance for PFS, OS, DMFS, and LRRFS. Therefore, the risk cluster we defined was more conducive to screening out the groups with poor sensitivity to therapy and an elevated risk of therapeutic failure during mid‐term treatment. In addition, the efficacy of each risk cluster correlated with distinct therapeutic regimens, facilitating the adjustment of therapeutic strategies for NPC patients and provide a basis for improving individualized precision treatment.

Multiple studies on the clinical types of LA‐NPC based on the biological behavior of tumors have demonstrated substantial variations in the clinical characteristics and survival outcomes among different clinical types of NPC.^7^, ^16^, ^17^, ^18^ In this work, the recurrence rate of type A NPC patients was 11.5% (16/139), slightly lower than the 11.6% (27/231) of type D NPC patients. However, the local recurrence rate of type A NPC patients was 10.8% (15/139), and the regional recurrence rate was 0.7% (1/139), while the local recurrence rate of type D NPC was 9.0% (21/231), accompained by a regional recurrence rate of 2.6% (6/231). The survival outcome aligned closely with previous studies.^19^ The proportion of NPC by clinical type varies from region to region, and Southern China is acknowledged as a high‐prevalence area.^7^, ^9^, ^19^, ^20^ The largest proportion of LA‐NPC is type AD, followed by type A, and type D is the least. Nonetheless, in our study, type AD accounted for the largest part of LA‐NPC, followed by type D and type A. One possible reason is that the patient population in this study is located in Southwest China, which is not a high‐incidence area, and people there have different living habits and genes, resulting in different proportions of clinical types.^21^, ^22^

The TVRR has been proven to hold prognostic value across a variety of solid tumors.^23^, ^24^ However, the mechanism underlying its impact on the prognosis remains unclear. In the process of radiotherapy‐dominated therapy for tumors, TVRR reflects the sensitivity of radiotherapy. This may be due to the tumor's inherent sensitivity to radiation, the proportion of hypoxic cells, and the tumor's ability to repair radiation injury.^25^ Grossman et al.^26^ were the first to apply TVRR to evaluate the efficacy of solid tumors during treatment to guide the following treatment. Nevertheless, relying solely on palpation and inspection to evaluate TVRR introduces an excessive degree of subjective bias into the results. To improve the precision of tumor volume assessment, scholars have launched studies on TVRR by calculating based on CT imaging examination in solid tumors.^12^ Although the use of CT reconstruction to extract tumor volume has improved the accuracy of calculating tumor regression rates, CT is comparatively less adept distinguishing soft tissues compared with MRI.^27^, ^28^ Currently, MRI examination is used for evaluating TVRR in various cancers, and the results in turn verify the excellence of MRI. For example, Antoine^29^ employed MRI to evaluate the TVRR after radiotherapy and chemotherapy, and the results indicated that the level of TVRR was the basis for adjusting the radiation dose. As far as we know, no scholar is using MRI to assess TVRR during radiotherapy for NPC. Therefore, this study matched MRI and CT images, manifesting a discernible enhancement in target area delineation, rendering the calculation of volume parameters and TVRR more dependable, and consequently, bolstering the credibility of the analytical outcomes.^30^

To effectively identify NPC patients exhibiting poor treatment effects and a high‐risk of failure, a reasonable risk stratification is required. Thus, researchers have conducted related studies. In the study of He et al.^31^ risk stratification of peripheral nerve invasion (PNI) and age before treatment demonstrated that NPC patients treated with NACT+CCRT in the low PNI and advanced age group have the improved predictive ability of OS and FPS indeed. Earlier research by Chen^32^ indicated that combined factors (higher N stage, higher level of serum ferritin, and lower level of serum albumin) might predict the ability of distant metastasis after standardized treatment of NPC. It is worth noting that both aforementioned studies exploring NPC prognosis based on the combination of pre‐treatment clinical characteristics and test indicators were carried out in non‐endemic areas in China, and notably, did not include EBV DNA titers. This omission may be attributed to different prognostic factors caused by different regions of NPC balF2‐CCT gene infection rate of EBV virus and RPMS1 gene variation.^33^, ^34^ Thus, this is one of the reasons why EBV DNA titers were not included in our research. Relevant studies have also been performed in areas where NPC is endemic in Southern China,^35^, ^36^ and it has been observed that the combination of tumor volume and clinical indicators could predict prognosis and guide subsequent treatment.^37^ Jeremy et al.^38^ established a prognostic scoring system incorporating clinical indicators, underscoring the feasibility of risk stratification for metastatic NPC patients. Notably, patients with low‐risk scores are likely to obtain good metastatic survival. Cumulatively, the results of these studies indicate that the combination of clinical features, imaging, tumor volume, and other factors can assess the risk of NPC prognosis. However, their stratification did not contain the important factor of tumor sensitivity to treatment, and their research lacked a comparison of performance in different risk stratification methods before treatment. Due to the lack of studies on mid‐term risk stratification of radiotherapy and chemotherapy, this work carried out risk stratification for the mid‐term treatment of NPC. Considering the early sensitivity of nasopharyngeal tumors to radiotherapy, the stratification factors included clinical classification and TVRR during treatment. We found differences survival rates in G1–6 groups, among them, G3 and G5 groups shows the worst prognosis for DMFS particularly, which may be due to the presence of large or multi‐station metastatic lymph nodes, making the tumor more inherently biologic aggressive. The multivariate analysis revealed that the risk cluster we defined during chemoradiotherapy was an independent prognostic factor for PFS, OS, DMFS, and LRRFS. This cluster facilitates the identification of NPC patients exhibiting poor therapeutic responses, thereby enabling clinicians to timely adjust treatment decisions.

Currently, the TNM staging system is an essential tool for predicting tumor prognosis.^5^ Therefore, we conducted a comparison between the prognostic performance of the TNM staging system and the risk stratification defined by us during radiotherapy and chemotherapy. Our findings suggest that the risk stratification was superior to the TNM staging system in predicting PFS, OS, DMFS, and LRRFS. Furthermore, we performed subgroup analyses on the risk cluster, revealing a markedly diminished survival rate within the high‐risk cluster in comparison to the low‐ and intermediate‐risk clusters. Thus, it is vital to adjust the therapeutic schedule and improve the survival rate of the high‐risk group after the risk assessment during chemoradiotherapy. Du et al.^39^ analyzed the therapeutic regimen for high‐risk groups of NPC and found that high‐risk patients treated with NACT + CCRT had better PFS and DMFS than those treated with CCRT, which is consistent with our results. Nonetheless, there is still controversy regarding whether ACT is useful for improving the survival rate of NPC. For instance, Liang et al.^40^ found that ACT did not improve the prognosis when evaluating the efficacy of CCRT versus CCRT+ACT for LA‐NPC. In contrast, Zhong et al.^41^ observed that CCRT+ACT was better than CCRT. Tang et al.^42^ conducted a propensity matching analysis on 550 NPC patients and found that NACT+CCRT has similar efficacy to CCRT+ACT, which is in line with our results. This study showed that after stratification during chemoradiotherapy, there was no difference in the three treatment methods in the low‐risk cluster, and the intensity of treatment should be reduced to lessen the toxic and side effects in the mid‐term treatment. Additionally, more research is needed for patients in the intermediate‐risk cluster to improve the survival rate, and ACT may be considered for patients in the high‐risk cluster to improve the survival rate in the later period.

This study may have some limitations. First, it is retrospective in nature, and future prospective studies are necessary to validate our findings. However, the clinical data and the follow‐up information we collected were true and reliable, and the data preservation was completed to ensure the objectivity and authenticity of our results. Second, being a single‐center study conducted in a non‐endemic area of China, further multi‐center validations, including endemic areas, are needed.

This work investigated the correlation between TVRR and prognostic value in 605 cases with different clinical stages of LA‐NPC during chemoradiotherapy. Our results indicated that clinical types and TVRR facilitate risk stratification for NPC during chemoradiotherapy, enabling clinicians to assess NPC patients with poor therapeutic effect and high risk of failure after treatment. This, in turn, provides a basis for adjusting therapeutic regimens. Moreover, the risk cluster we defined for NPC patients during the mid‐term of chemoradiotherapy serves as an independent prognostic factor for PFS, OS, DMFS, and LRRFS after treatment, and shows great capacity in predicting PFS, OS, DMFS, and LRRFS. In addition, NPC patients in the high‐risk cluster may benefit from ACT following CCRT.

## 5 CONCLUSIONS

The reliance on clinical types and TVRR facilitates risk stratification of NPC during chemoradiotherapy, providing a foundation for physicians to tailor therapeutic strategies. Moreover, the risk cluster delineated for NPC patients during the mid‐term of chemoradiotherapy stands as an independent prognostic factor for PFS, OS, DMFS, and LRRFS posttreatment. Additionally, individuals in the high‐risk cluster are recommended to undergo ACT after CCRT.

## AUTHOR CONTRIBUTIONS

**QianLong Tang:** Conceptualization (equal); data curation (equal); formal analysis (equal); investigation (equal); methodology (equal); resources (equal); software (equal); validation (equal); visualization (equal); writing – original draft (equal). **Chaorong Mei:** Conceptualization (equal); data curation (equal); formal analysis (equal); funding acquisition (equal); investigation (equal); methodology (equal); project administration (equal); resources (equal); software (equal); writing – original draft (equal). **Bei Huang:** Conceptualization (equal); data curation (equal); formal analysis (equal); investigation (equal); methodology (equal); resources (equal). **Rui Huang:** Conceptualization (equal); data curation (equal); investigation (equal); resources (equal); software (equal). **Le Kang:** Data curation (equal); formal analysis (equal); investigation (equal); resources (equal). **Ailin Chen:** Data curation (equal); formal analysis (equal); funding acquisition (equal); investigation (equal). **Na Lei:** Data curation (equal); formal analysis (equal). **Pengcheng Deng:** Data curation (equal); formal analysis (equal); investigation (equal); resources (equal). **Shouyan Ying:** Data curation (equal); formal analysis (equal); investigation (equal). **Peng Zhang:** Conceptualization (equal); data curation (equal); formal analysis (equal); funding acquisition (equal); investigation (equal); methodology (equal); project administration (equal); resources (equal); software (equal); supervision (equal); validation (equal); visualization (equal); writing – review and editing (equal). **Yuan Qin:** Conceptualization (equal); data curation (equal); formal analysis (equal); investigation (equal); methodology (equal); project administration (equal); resources (equal); software (equal); supervision (equal); validation (equal); visualization (equal); writing – review and editing (equal).

## CONFLICT OF INTEREST STATEMENT

The authors declare that there are no financial and personal relationships with other people or organizations that can inappropriately influence this work. There is no professional or other personal interest of any nature or kind in any service and/or company that could be construed as influencing the position presented in, or the review of, the manuscript entitled. The submission of the article implies that the work described has not been published previously and is not under consideration for publication elsewhere.

## FUNDING SUPPORT

This study was supported by the natural science foundation of Tibetan Autonomous Region (XZ202001ZR0064G).

## ETHICS STATEMENT

This study was approved by the Ethics Committee of Medical Research and New Medical Technology of Sichuan Cancer Hospital (SCCHEC‐02‐2022‐160).

## Supporting information


Data S1.

## Data Availability

The authors declare that there are no financial and personal relationships with other people or organizations that can inappropriatelyinfluence this work. There is no professional or other personal interest of any nature or kind in any service and/or company that couldbe construed as influencing the position presented in, or the reviewof, the manuscript entitled. The submission of the article implies thatthe work described has not been published previously and is notunder consideration for publication elsewhere.
