# Supplementary material for: Risk stratification of LA‐NPC during chemoradiotherapy based on clinical classification and TVRR
Source: Cancer Med. 2024 Feb 23;13(3):e7029. doi: 10.1002/cam4.7029 (PMC10891362; doi:10.1002/cam4.7029)

**LIST OF SUPPORTING INFORMATION**

Supporting Table 1. Survival outcomes for patients with locoregionally advanced nasopharyngeal carcinoma in six groups classified by three clinical types combined with the tumor volume reduction rate (TVRR) during definitive chemoradiotherapy.

| Groups | 5-year PFS | 5-year OS | 5-year DMFS | 5-year LRRFS |
| --- | --- | --- | --- | --- |
| G1 | 53.7% | 66.7% | 87.0% | 75.9% |
| G2 | 92.9% | 95.3% | 97.6% | 97.6% |
| G3 | 43.9% | 68.3% | 64.6% | 81.7% |
| G4 | 87.2% | 95.3% | 92.6% | 94.6% |
| G5 | 32.0% | 66.7% | 64.0% | 80.0% |
| G6 | 69.4% | 81.8% | 81.9% | 91.2% |
| *P* values | < 0.001 | < 0.001 | < 0.001 | < 0.001 |

Supporting Table 2. The number of events between three distinct risk clusters.

| **Number of events** | **Low-risk cluster**  **(n = 234, %)** | **Intermediate-risk cluster**  **(n = 214, %)** | **High-risk cluster**  **(n = 157, %)** |
| --- | --- | --- | --- |
| **Local-regional recurrence** | 12 (5.1) | 30 (14.0) | 34 (21.6) |
| **distant metastasis** | 21 (8.9) | 41 (19.1) | 66 (42.0) |

**Low–risk cluster:** The type A NPC with high TVRR (TVRR≥50%) and the type D NPC with high TVRR (TVRR≥50%); **Intermediate–risk cluster:** The type A NPC (T3–4N0–1) with low TVRR (TVRR<50%) and the type AD NPC with high TVRR (TVRR<50%); **High–risk cluster:** The type D NPC (T1-2N2–3) with low TVRR (TVRR<50%) and the type AD NPC (T3–4N2–3) with low TVRR (TVRR≥50%).

Supporting Table 3. Survival outcomes among three distinct risk clusters.

| **Clusters** | **5-year PFS** | **5-year OS** | **5-year DMFS** | **5-year LRRFS** |
| --- | --- | --- | --- | --- |
| **Low-risk cluster** | 89.3% | 95.3% | 94.4% | 96.5% |
| **Intermediate-risk cluster** | 65.4% | 78.0% | 83.2% | 87.4% |
| **High-risk cluster** | 38.2% | 67.5% | 64.3% | 80.9% |
| ***P* values** | < 0.001 | < 0.001 | < 0.001 | < 0.001 |

Supporting Table 4. Comparison of prognostic performance between risk stratification system and TNM staging system.

|  | **C-index (95% CI)** | **AUC values** |
| --- | --- | --- |
| PFS |  |  |
| The risk clusters | 0.751 (0.713-0.789) | 0.746 |
| TNM stage | 0.597 (0.556-0.611) | 0.617 |
| OS |  |  |
| The risk clusters | 0.712 (0.667-0.757) | 0.713 |
| TNM stage | 0.604 (0.557-0.651) | 0.647 |
| DMFS |  |  |
| The risk clusters | 0.704 (0.655-0.753) | 0.744 |
| TNM stage | 0.554 (0.505-0.603) | 0.594 |
| LRRFS |  |  |
| The risk clusters | 0.659 (0.599-0.719) | 0.705 |
| TNM stage | 0.513 (0.452-0.574) | 0.557 |

Supporting Figure 1. The summary of multivariate Cox proportional hazard regression analysis of independent prognostic factors for 605 LA-NPCs.


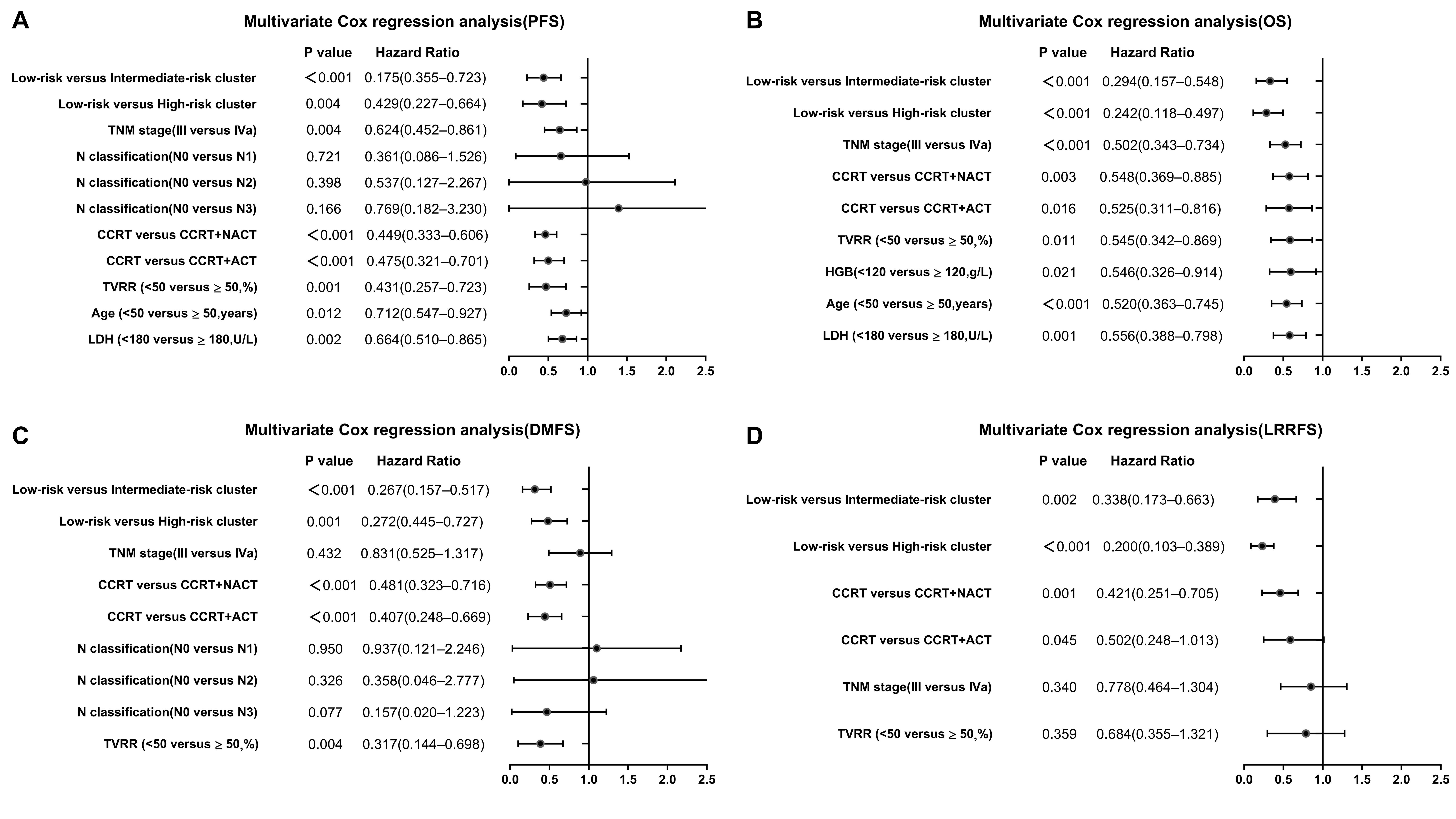


Supporting Figure 2. The summary of multivariate Cox proportional hazard regression analysis of independent prognostic factors for the high-risk cluster.


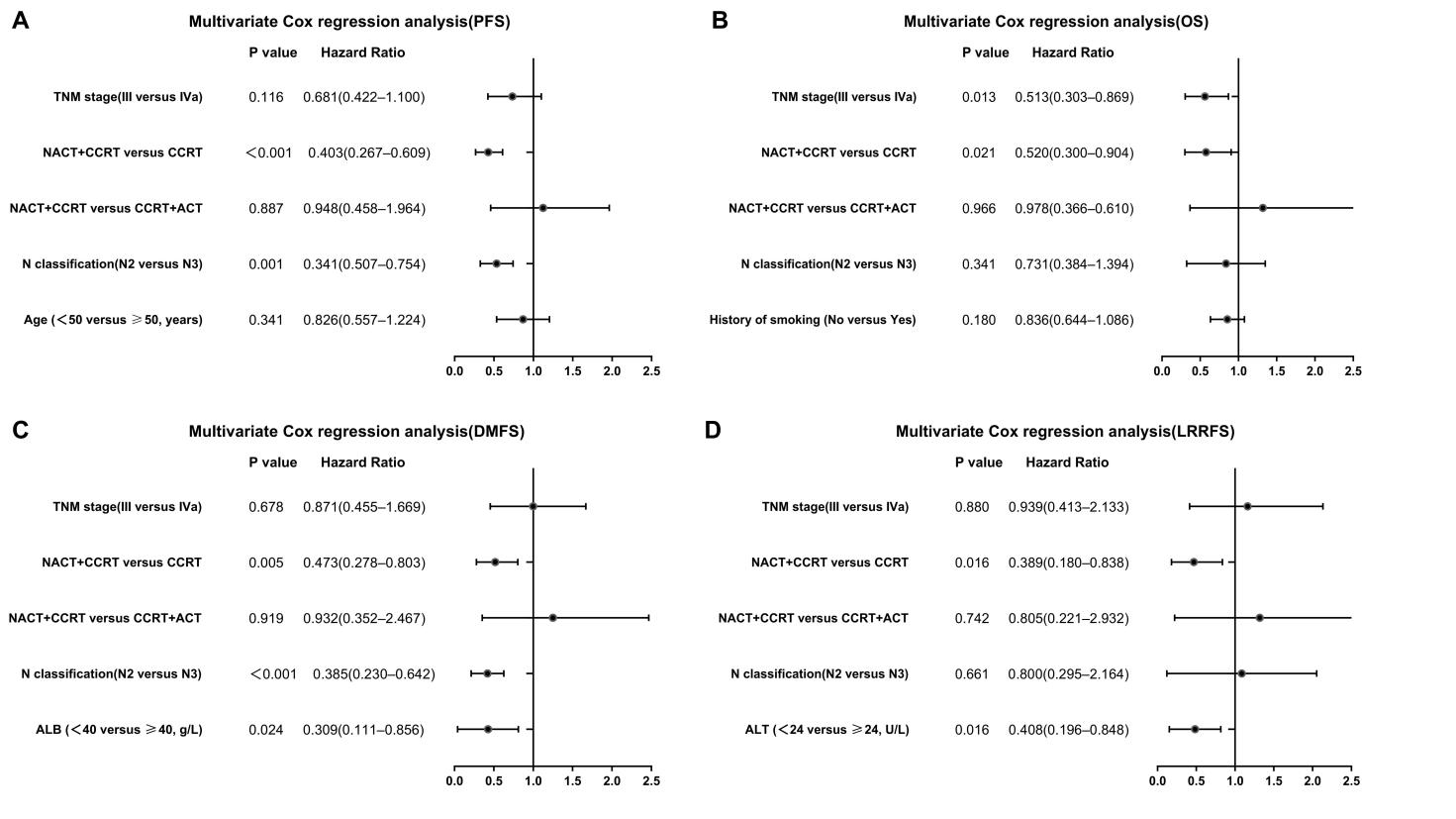

Supplement: Supplementary file 1 — Data S1. [file CAM4-13-e7029-s001.docx]
